# Effects of Effective Microorganisms on Meat Quality, Microstructure of the *Longissimus Lumborum* Muscle, and Electrophoretic Protein Separation in Pigs Fed on Different Diets

**DOI:** 10.3390/ani10101755

**Published:** 2020-09-26

**Authors:** Patrycja Reszka, Dorota Cygan-Szczegielniak, Hanna Jankowiak, Aleksandra Cebulska, Beata Mikołajczak, Joanna Bogucka

**Affiliations:** 1 Department of Animal Physiology, Physiotherapy and Nutrition, Faculty of Animal Breeding and Biology, UTP University of Science and Technology, Mazowiecka 28, 85-084 Bydgoszcz, Poland; cygan@utp.edu.pl (D.C.-S.); bogucka@utp.edu.pl (J.B.); 2 Department of Animal Breeding, Faculty of Animal Breeding and Biology, UTP University of Science and Technology, Mazowiecka 28, 85-004 Bydgoszcz, Poland; jankowiak@utp.edu.pl (H.J.); cebulska@utp.edu.pl (A.C.); 3 Department of Meat Technology, Faculty of Food Science and Nutrition, Poznań University of Life Sciences, Wojska Polskiego 31, 60-624 Poznań, Poland; beata.mikolajczak@up.poznan.pl

**Keywords:** pigs, probiotic, meat quality, muscle fibers, histopathological parameters, intramuscular fat, protein electrophoresis

## Abstract

**Simple Summary:**

Pork is the most popular meat among consumers in Poland, but it can also be a source of pathogens. Therefore, there is a need to find effective prophylaxis in order to ensure that consumers have access to safe food with the desired nutritional qualities and, above all, food that is free of pathogens. In order to meet these expectations, producers use probiotics containing effective microorganisms (EMs) offered in the market. In this study the same probiotic, EM Bokashi, was used for the first time in combination with three nutritional variants with proteins of different origin.

**Abstract:**

The aim of the study was to determine how effective microorganisms influence meat quality, the microstructure of the longissimus lumborum muscle, and electrophoretic protein separation. The study group consisted of 150 piglets divided into three feeding groups: C, E1, and E2. The feeding groups included C—a standard fodder blend with a full share of post-extracted soya meal; E1—a 50%/50% mix of pea and lupine/soya bean in phase I of fattening and a 75%/25% mix of pea and lupine/soya bean in phase II of fattening; and E2—a 50%/50% mix of pea and lupine/soya bean in phase I of fattening and in 100% pea and lupine in phase II of fattening. The experimental factor was the addition of the EM Carbon Bokashi probiotic to the diet (C + EM, E1 + EM, E2 + EM). Influence of the feeding system on the following parameters was also estimated. After slaughter, the meat quality, *LL* muscle microstructure, and electrophoretic protein separation were assessed. In the C + EM group, a lower water-holding capacity was demonstrated. Meat from pigs fed the effective microorganism additive was much harder in the E1+EM group compared to meat from pigs from the E1 group. A beneficial effect of effective microorganism was found in the E2 + EM group, where less thermal leakage from the meat was demonstrated. A beneficial effect of the feeding system on thermal leakage and loin eye area in the E2 + EM group was demonstrated. In the C + EM group, a lower total number of muscle fibers was demonstrated. The addition of effective microorganism caused an increase in the diameter of fast twitch fibers in the E1 + EM group. In the same group of pigs, effective microorganisms caused a lower proportion of fiber fission. This nutritional variant appears to be the most appropriate for proteins as well, because it led to the most favorable percentage of individual proteins after effective microorganisms supplementation in the longissimus lumborum muscle.

## 1. Introduction

Pork is one of the most popular meat choices for consumers in worldwide [1]. Unfortunately, it is also often a potential source of pathogens that occur naturally in pigs but cause diseases due to microorganisms such as *Salmonella* spp., *Toxoplasma gondii*, *Listeria monocytogenes*, *Campylobacter* spp., and *Staphylococcus aureus*. In order to develop an effective prophylaxis of pathogen-free pork consumption, it is important to find appropriate methods to eliminate pathogens from meat [2]. Healthy food with appropriate dietary qualities is a priority among consumers. Therefore, in order to meet these expectations, producers use feed additives—probiotics—containing effective microorganisms (EMs), which combat pathogenic microflora and improve the microbiological balance of the digestive system and meat quality [3,4]. Many authors have shown a beneficial effect of probiotic preparations on meat quality, including succulence, elasticity, tenderness, and color [3,4,5]. On the other hand, intensive breeding work, aimed at achieving the highest possible production—and, thus, the highest meat content of fattening pigs—has led to histopathological changes in the muscles, such as changes in the size and shape of fibers (atrophy or hypertrophy), splitting, and fiber necrosis. The individuals in which we most frequently encounter these changes are fattening pigs of the pietrain breed and its hybrids [6]. According to the existing research, intensive selection, in addition to genetic conditions, nutrition, body weight, and breed, may also affect the number of muscle fibers [7]. The histological and biochemical properties of the muscle, e.g., the area and type of fibers or the glycolytic and oxidative properties, are the elements that determine the quality of meat [8,9].

In January 2006, after a ban on antibiotics was introduced in the European Union, all dietary supplements (e.g., probiotics, prebiotics, synbiotics, and herbs) have become an object of interest for scientists and the subject of many studies in order to find an effective alternative. The term ‘probiotic’ refers to live microorganisms (with proven probiotic properties and origin) that, when administered in sufficient quantities, have a beneficial effect on animal health and productivity [10]. Currently, the majority of the produced probiotics use lactic acid bacteria (*Lactobacillus acidophilus*), which prevent the development of *Salmonella*, *Escherichia coli*, and *Candida albicans* and stimulate immunity. Other strains of the genus *Lactobacillus* synthesize vitamins D and K (*L. brevis*), produce the antibiotic lactolin (*L. plantrum*), or increase tolerance to lactose (*L. rhamnosus*). Other bacteria used in probiotic production, such as *Bifidobacterium bifidium*, inhibit the development of pathogens; *Enterococcus faecium* destroys rotaviruses [11]. The probiotic preparation used in this experiment contains strains of microorganisms, including *Lactobacillus plantrum*, *Enterococcus faecium*, *Bacillus licheniformis*, and *Clostridium butyricum*, which do not undergo any technological treatment [12].

In Poland, where 80% of animal nutrition is based on imported soya bean meal, it is important to find an alternative source of protein for reasons of the food safety of both humans and animals. The solution to this problem may be to use native legumes—peas and lupine [13]. Therefore, this paper presents three nutritional variants, with proteins of different origin, combined with the addition of a probiotic.

The aim of the study was to determine how effective microorganisms (EM) influence meat quality, the microstructure of the longissimus lumborum (*LL*) muscle (the enzymatic profile of the muscle fibers, intramuscular fat content, and the occurrence of pathological changes), and the electrophoretic distribution of selected proteins in pigs fed on diets with proteins of different origin.

## 2. Materials and Methods

### 2.1. Maintenance and Feeding of Animals

The tests were carried out at the Fatstock Pig Utility Control Station in Mełno, Poland. The study covered 150 piglets ♀F1 (Polish Landrace PL × Polish Large White PLW) with ♂F1 (Pietrain × Duroc), 50% gilts and 50% hogs), at an initial body weight of about 30 kg. The animals were placed into two separate, identically constructed and equipped rooms—the control room (groups C, E1, and E2) and the experimental room (groups C + EM, E1 + EM, and E2 + EM). Each animal was marked and placed in an individual 2 m^2^ pen with straw bedding equipped with automatic feeder and nipple drinker, allowing constant access to water (Directive No. 2010/63/EU). Wheat straw was replaced three times a week and animals’ pens were augmented by metal chains.

The fattening pigs were divided into three groups: the control group (C) and the experimental groups (E1 and E2), depending on the nutrition they were receiving. Group C was fed a standard diet based on post-extraction soya bean meal (100%). In fattening phase I, groups E1 and E2 were given a feed mixture with 50% soya bean and 50% pea and lupine. In fattening phase II, group E1 were fed a compound feed of 25% soya bean and 75% pea and lupine, while in group E2 soya bean protein was completely replaced with 100% pea and lupine. The composition of the feeds is shown in Table 1.

In order to assess the effect of the probiotic used, animals were divided into control subgroups (without the probiotic)—C, E1, and E2—and experimental subgroups (with the probiotic)—C + EM, E1 + EM, and E2 + EM (Table 2).

The laboratory assessment covered meat samples taken from 60 animals (10 randomly pigs from each group: five gilts and five hogs) which were pooled into a single study group within each feeding system because there were no significant sex-related differences in the values of the analyzed parameters (Table 2).

The applied probiotic EM Carbon Bokashi [14] was administered in the amount of 5 kg per tonne of feed (in phase I of fattening) and 3 kg per tonne of feed (in phase II of fattening). Animals weighing approximately 115 kg were slaughtered in accordance with the applicable standards and regulations (Ordinance of the Minister of Agriculture and Rural Development of 9 September 2004). An electric stunning method was applied to the pigs.

### 2.2. Quality of Meat

The acidification of muscle tissue 48 h after slaughter (pH_48_) was determined using an Elmetron CP-401 pH meter with a firing electrode. The equipment was calibrated using Elmetron’s pH 7.0 and pH 4.0 buffers. Meat quality was assessed 48 h after slaughter (without repetitions) on the *LL* muscle, which was stored at 4–6 °C. The water-holding capacity (WHC) was determined according to Grau and Hamm [15], as modified by Pohji and Ninnivaara [16]. A 300-mg sample of minced meat was weighed and placed on Whatman filter paper between two glass plates under a constant load of 2 kg for 5 min. The juice infiltration area was used to calculate the percentage of loose water in the meat, assuming that 1 cm^2^ of infiltration corresponds to 10 mg of water. The infiltration surface of meat juice was measured with a planimeter. WHC was reported as the mean of three trials.

The thermal leakage was determined according to the method developed by Walczak [17], without repetitions. A 20-mg meat sample was placed on a hygroscopic gauze and heated at 85 °C for 10 min in a water bath. After heating and removing the gauze, the sample was cooled to 4 °C and weighed. On the basis of the weight difference before and after heat treatment, the percentage of weight loss was calculated.

The color of the meat was measured on a slice of raw meat using a Minolta CR 310 photocolorimeter (Konica Minolta, Tokyo, Japan) with a measuring hole diameter of 50 mm. The instrument was standardised using the white calibration plate CR310 with coordinates Y = 92.80, x = 0.3175 and y = 0.3333. The color parameters of individual samples were determined in the CIE L*a*b* system (L*—brightness; a*—proportion of red; b*—proportion of yellow) [18] using Illuminant D65 and a standard 2° observer, without repetitions.

Marbling was determined using a slice of raw meat weighing 120 g. The visual assessment was carried out by a team of five specialists and the results was presented as the mean of these five assessments. Marbling was assessed on the basis of Canadian and American models on a 10-degree scale [19,20], where 1 = no intramuscular fat content and 10 = very high marbling.

The tenderness of the meat was determined using an Instron 3342 strength tester equipped with a Warner-Bratzler cap, according to the method by Szalata et al. [21]. Meat samples weighing 120 g were heated in a water bath to an internal temperature of 70 °C. Heat treatment was conducted in a 0.85% NaCl solution. Five 10 mm × 10 mm rods were cut out along the muscle fibers, then cut perpendicularly to the muscle fibers. The result, which is the mean of five trials, was recorded as maximum shear force expressed in N.

The outline of the surface area of the *LL* muscle (sirloin eye area) was drawn on a slice of raw meat (120 g) and the surface area (cm^2^) was determined using the planimetric method.

### 2.3. Histochemical Analysis

To assess the microstructure of the muscle, a 5 × 15 mm sample was taken from the longissimus lubmorum (*LL*) muscle between the 4th and 5th ribs. Next, the samples were frozen in liquid nitrogen at −196 °C. The frozen muscle samples were transferred to cryostat (Thermo Shandon, London, UK) and cut into 10-μm thick histological fragments at about −25 °C. The fragments were then placed on a slide and subjected to the following histochemical reactions:

In order to determine the muscle fiber types, a combined reaction was carried out to the activity of two enzymes [22]: tetrazoline reductase NADH-TR (incubation of preparations in the incubation fluid: NADH, NBT, and 0.1M phosphate buffer at pH 7.4 and 37 °C for 1 h) and myofibrillar ATP (pre-incubation of the preparations in an acidic solution at pH 4.0 for 3 min, followed by incubation in an incubation fluid: ATP, CaCl_2_, and sodium barbiturate at pH 9.6 and 37 °C for 30 min). The effect of the reaction is the transformation of colorless tetrazolium salts into visible formazan granules, which allows for an unambiguous determination of the metabolic/structural type of fibers: STO (slow twitch oxidative fiber, dark brown or black), FTO (fast twitch oxidative fiber, blue), and FTG (fast twitch glycolytic fiber, light, straw-colored).

In order to determine the percentage of intramuscular fat (IMF; intramuscular fat is red), the samples were dyed oil red according to Dubovitz et al. [23]. First, the slides was taken from the freezer and left to dry at room temperature for two minutes. Then, immersing the slides in various reagents with specific periods of time: formaldehyde 4% (5 min), distilled water (5 min), oil red solution (Sigma-Aldrich, Poznan, PL, 15 min), distilled water (15 min).

In order to determine pathological changes in the muscle, topographic staining with hematoxylin and eosin was performed. Pathological changes, such as giant fibers, necrotic fibers, and splitting, were determined.

Then, microscopic images were recorded on a computer disk using a Nikon Ci-L microscope equipped with a Nikon DS-Fi3 camera with a resolution of 5.9 MPix and NIS ELEMENTS software, enabling linear and planimetric measurements and digital analysis of the microscopic images. The percentage of individual muscle fiber types was calculated for an area of 1.5 mm^2^. The diameters of muscle fibers were measured according to the method given by Brooke [24]: measuring the shortest diameters. The density of muscle fibers was calculated based on the average number of fibers per 1.5 mm^2^. The total number of fibers (TNF) was calculated by multiplying the fiber density and the sirloin eye area. Intramuscular fat (IMF) was determined over an area of 3 mm^2^ using the function of filtering out the indicated color, which calculates the percentage of red. The software also calculated the number of normal and pathological fibers over the area of 1.5 mm^2^ and the percentage content of these fibers.

### 2.4. Sodium Dodecyl Sulfate-Polyacrylamide Gel Electrophoresis (SDS-PAGE)

#### 2.4.1. Sample Preparation

Samples of 2 mg of raw frozen meat were mixed with a 98-µL buffer (pH 6.8; 8 M urea, 2 M thiourea, 0.05 M Tris–HCl, 0.075 M DTT, 3% [*w*/*v*] SDS, 0.05% [*w*/*v*] bromophenol blue). Each sample was heated for 3 min at 98 °C. The protein concentration in 10-µL samples was determined using a 2-D Quant Kit (GE Healthcare Bio-Sciences, Marlborough, MA, USA). Protein from each sample (20 μg) was subjected to electrophoresis.

#### 2.4.2. Gel Electrophoresis

The proteins were separated using SDS-PAGE in 15% polyacrylamide two-phase gels [25]. The resolving gel contained 30% (*w*/*v*) acrylamide, 75% (*v*/*v*) glycerol, 3 M Tris [pH 8.8], 10% (*w*/*v*) SDS, 1% (*w*/*v*) ammonium persulfate, and 16 µL of TEMED. The stacking gel (10% (*w*/*v*) acrylamide, 5% (*v*/*v*) glycerol, 0.125 M Tris (pH 6.8), distilled water, 10% (*w*/*v*) SDS, 1% (*w*/*v*) ammonium persulfate, staining buffer, and 25 µL of TEMED) was poured on the layer of resolving gel. The separation was performed with buffer (0.25 M Tris base, 1.92 M glycine, 0.5% SDS (*w*/*v*), and 10 mM of 2-mercaptoethanol). A PageRuler Plus Protein Ladder 10–250 kDa (Thermo Fisher Scientific, Waltham, MA, USA) was used as a standard for protein molecular weight (m.w.) calibration. The gels were prepared in triplicate on 80 × 100 mm plates with 0.75-mm spacers. The separation was conducted using an SE 250 type apparatus (Hoefer Scientific Instruments Company, Holliston, MA, USA). Electrophoresis was run with a constant current of 40 mA per two gels. Proteins were visualized by staining with 0.05% (*w*/*v*) Coomassie Brilliant Blue R-250, 50% (*v*/*v*) methanol, and 10% (*v*/*v*) acetic acid for 0.5 h and then destained overnight (10% (*v*/*v*) methanol and 4.5% (*v*/*v*) acetic acid).

#### 2.4.3. Image Analysis

All images of the polyacrylamide gels were acquired using an Image Master^®^ VDS imaging system (Pharmacia Biotech, Vienna, Austria) and analyzed using Image Master^®^ 1D Elite v. 4.0 software. Computations were based on the assumption that the area of a single protein band accounts for a percentage ratio in relation to the area of all separated protein bands, which constitutes 100%.

### 2.5. Statistical Analysis

Since some results did not meet the assumptions of a normal distribution (which was verified using the Shapiro–Wilk test) or the assumptions of homoscedasticity—required for implementing parametric tests-the non-parametric Mann–Whitney U test was used in order to examine the statistically significant differences between two experimental groups within one feeding system (C vs. C + EM; E1 vs. E1 + EM and E2 vs. E2 + EM) for percentage of muscle fibers FTO and FTG, total number of muscle fibers (TNF), normal fibers (%), giant fibers (%), fiber necrosis (%), fiber splitting (%), intramuscular fat content (%), pH_48_, WHC (%) and for marbling score. For the remaining parameters which met the assumptions of a normal distribution and homogeneity of variance, the parametric analysis of variance (ANOVA) was applied, followed by a test for multiple comparisons, i.e., post hoc Tukey test (HSD). For the purpose of analyzing the relationships between selected parameters, Spearman’s rank correlation coefficient was applied. Additionally, the Kruskal–Wallis test and the comparison of mean ranks were used to test the differences between the feeding systems (C vs. E1 vs. E2 and C + EM vs. E1 + EM vs. E2 + EM). The obtained results were processed statistically using Statistica 13.1 software.

## 3. Results and Discussion

### 3.1. Quality of Meat

Table 3 presents the results of the meat quality assessment.

In control group C and experimental groups E1 and E2, no influence was demonstrated of feeding pigs effective microorganisms on the degree of the acidification of muscle tissue evaluated 48 h after slaughter. Previous research [3,26] also did not report an effect on pH_48_ from feeding fattening pigs effective microorganisms. Additionally, the studies conducted by Yang et al. [26] on broilers did not show the effect of *Clostridium butyricum* on the ultimate pH. In contrast, studies by Zhang et al. [9] showed an increase in pH_24_ meat of pigs after dietary supplementation with resveratrol—a flavonoid with antioxidant and anti-inflammatory effects—in the amount of 300 mg/L per kg of feed, compared to the control group (*p* < 0.05). Ultimate pH has a significant effect on WHC of meat. The higher ultimate pH might lead to the stronger WHC and the lower moisture losses [27]. In addition, the pH value has a significant impact on meat color and tenderness [28].

In the present study, meat from the experimental group C + EM—fed a standard diet with post-extraction soya bean meal with the addition of effective microorganisms—had lower WHC (18.16%) than the control group (15.57%) (*p* < 0.05). A study by Dowarah et al. [29] reported improvement of WHC after supplementation with *Pediococcus acidilactis FT28* (*p* < 0.05). Chang et al. [28] did not demonstrate an effect of the probiotic on WHC. High WHC values may indicate a slow and steady rate of pH drop during the initial hours after slaughter [30]. WHC is an important criterion for classifying meat quality, mainly from the producer’s point of view, as it indicates the meat’s suitability for further processing, while, from the consumer’s point of view, it impacts on the culinary use of the meat [31]. The beneficial effect of effective microorganisms on thermal leakage was demonstrated in experimental group E2 + EM: it was statistically significantly lower compared to the control group (*p* < 0.05). Jukna et al. [3] found that after the addition of the probiotics Yeasture and Microbond to pig feed, an improvement in the culinary properties of the meat was found. Also, the influence of diet on thermal leakage was demonstrated. In the experimental group E1+EM thermal leakage was significantly higher, compared to group E2 + EM (*p* ≤ 0.05). Bocian et al. [32] showed no effect of feeding pigs with legumes on thermal leakage. Zmudzińska et al. [33] demonstrated that feeding pigs with diets containing legume plants and extracted rapeseed meal does not affect the pork meat quality. Other authors [34] showed that the experimental diet with an addition of 5% of raw soybean seeds affected (*p* < 0.05) meat color and also meat composition.

The application of the Bokashi preparation did not have a significant statistical effect on the change of meat color (L*a*b*) in the study groups. A study by Balasubramanian et al. [35] also showed no effect of the addition of the probiotic on meat color (L*: 58.42; a*: 17.47; b*: 6.10 at a supplementation of 0.1 MSP g/kg). Other authors, on the other hand, noted a much higher proportion of yellow among the group of pigs fed the probiotic (8.98 vs. 9.48; *p* < 0.05) [18]. Meng et al. [5] demonstrated the beneficial effect of supplementing the diet of fattening pigs with *Bacillus subtilis* and *Clostridium butyricum* on the proportion of yellow (*p* < 0.01) and brightness (*p* < 0.05).

The use of the Bokashi preparation did not have a significant statistical effect on meat marbling. This is consistent with the results from Balasubramanian et al. [35], but different from Meng et al. [5]. Marbling is defined as the amount and spatial distribution of visible, white fat spots present in meat. The proper degree of marbling has a beneficial effect on the succulence, tenderness, and palatability of the meat [19].

Another feature analyzed was tenderness, which depends on the age and species of the pig, as well as on the procedures before and after slaughter, among other things. The degree of tenderness is influenced by the time and conditions in which the meat matures and the proteolytic activity during storage [36]. In the experimental group E1 + EM (50% soya bean and 50% pea and lupine in phase I of fattening, 25% soya bean and 75% pea and lupine in phase II of fattening), the differences in terms of tenderness were statistically significant. The meat from pigs fed effective microorganisms was significantly harder (56.27 N) than the meat from the E1 control group (47.47 N; *p* < 0.05). On the other hand, an experiment by Yang et al. [27] found reduced cutting force in poultry, which indicates improved tenderness. However, Chang et al. [28] and Liu et al. [37] did not demonstrate a significant effect of probiotic use on meat tenderness. The influence of diet on meat tenderness was also demonstrated, which differed statistically significantly between the experimental groups (C + EM, E1 + EM and E2 + EM) (*p* ≤ 0.05). The most desirable, tender meat was obtained from pigs from the C + EM group, while the hardest was obtained from pigs from the E1 + EM group. In the studies by Bocian et al. [32] showed no effect of feeding pigs with legumes on meat tenderness. No effect of pig feeding with Bokashi preparation on the sirloin eye area was demonstrated in this study. Study by Balasubramanian et al. [38] also reported no effect of supplementation of the probiotic preparation *Bacillus* spp. at the levels of 0.01% and 0.02% on the sirloin eye area (68.67 and 69.47 cm^2^, respectively). However, the influence of the feeding system on this feature was noted. Significantly larger sirloin eye area was demonstrated in pigs in the E2 + EM group, compared to E1 + EM (*p* ≤ 0.05). The effect of probiotics may vary, as it depends on the type of bacteria used, the level and time of supplementation, the composition of the diet, and interactions with other dietary supplements [5].

### 3.2. Microstructure of the Longissimus Lumborum Muscle

This is one of the few studies that deals with the influence of effective microorganisms on the microstructure of the *LL* muscle in pigs. The microstructure of the *LL* muscle and the proportion of pathological lesions is presented in Table 4.

**Figure 1:**
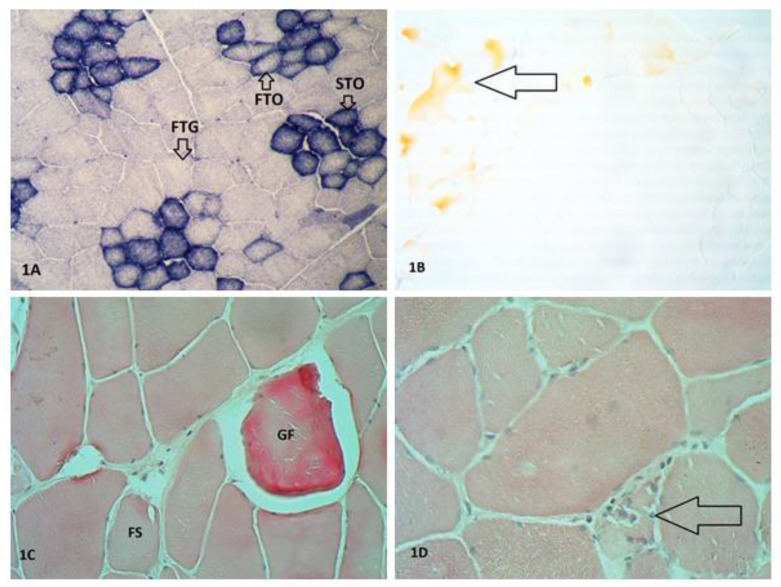
(**A**) muscle fiber types (group E1 + EM), NADH-TR tetrazolium reductase activity and myofibrillar ATP-ase activity stain, magnification ×125; (**B**) intramuscular fat (arrow) (group E2), red oil stain, magnification 125×; (**C**) giant fiber (GF) and fiber splitting (FS) (E1), haematoxylin and eosin (H&E) stain, magnification 250×; (**D**) fiber necrosis (group C + EM) (arrow) (C + EM), H&E stain, magnification 400×.

The proportions of different types of muscle fibers are one of the most important factors that influence the quality of meat and depend on the nutrition, breed, sex, age, and physical activity of the animal [7,39]. The study did not find any influence of effective microorganisms on the proportions of particular muscle fiber types (Figure 1A) and their density. Such an influence was demonstrated, however, by Lebedová et al. [40], who divided the animals into three groups: AL—ad libitum nutrition, R1—feed limitation, and R2—strong feed limitation. The mix from the AL group had a significantly lower proportion of FTG fibers (81.38%) compared to pigs from Groups R1 and R2 (84.03 and 84.18%, respectively; *p* < 0.05). Research by Brzobohaty et al. [41] carried out on hybrids of the D × genotype (LW_D_ × L) also found a significant effect of nutrition on the proportions of muscle fibers (*p* < 0.001). Pigs fed the highest level of rapeseed meal recorded the highest percentage of STO fibers (16%) and fiber density per mm^2^ (31). The group of animals receiving the lowest level of crude protein (142,82 g vs. 146,93 vs. 153,69 g) was characterised by the highest percentage of FTG fibers (86%), while the density of fibers was 22/mm^2^ [41]. The addition of resveratrolto the diet of fattening pigs at 300 and 600 mg/kg resulted in a change to more oxidative muscle fibers [9]. In this study a tendency towards fewer fibers per unit of muscle area and significant difference in TNF (*p* < 0.05) was observed in experimental groups C + EM, which affects the tender/fine structure of meat. In experiment was found a significantly larger diameter of FTO and FTG fibers in only the experimental group E1 with addition of effective microorganisms (*p* < 0.05). Of the muscle fiber types studied, the largest diameters were recorded for FTG fibers. Research by Zhou et al. [42] showed that the dietary supplementation of fattening pigs with 0.08% polyphenolic extract of *Eucommia ulmoides* Oliver (PEE) leaves resulted in a decrease in fiber diameter with increased fiber density (*p* < 0.05). A study by Muqader [43] reported larger diameters of muscle fibers in broiler chickens whose diet was supplemented with zinc. Similarly, a study by Zhou et al. [44] carried out on the thoracic and femoral muscles showed that the addition of 200 mg/kg and 400 mg/kg of probiotics (*Bacillus subtilis*, *Bacillus licheniformis*, and *Bacillus natto*) to the diet of broilers had a significant effect on the growth of muscle mass and the diameter of muscle fibers at a late stage of growth. Jin et al. [45], in their study on the thoracic and femoral muscles, found a reduction in the diameter of muscle fibers (*p* < 0.05) after the addition of 0.3 mg/kg of selenium yeast and 5 mg/kg of boron to the diet of chickens. After adding the a synbiotic consisting of 0.8% RFO prebiotic and 1% Lavipan^®^ probiotic for Ross 308 chicken feed, Bogucka et al. [46] also observed a tendency of reduced-diameter muscle fibers. This resulted in an increase in fiber density—the fineness of the meat—which had a beneficial effect on meat quality. Moreover, the influence of different diet on the diameter of FTG fibers was also demonstrated. They were significantly larger in the E2 group, in which the diet was based only on pea and lupine (100% in the II phase of fattening), compared to E1 group (respectively: 51.26 vs. 44,68 µm) (*p* < 0.05). Bogucka et al. [47] demonstrated the impact of different nutritional strategies on the histochemical characteristics of pig muscles. The authors was found that pigs receiving less protein and energy in their diet were characterized by a higher proportion and increased diameter of FTG fibers. A higher percentage of FTG fibers with limited nutrition was confirmed by Skiba et al. [48] and Brzobohaty et al. [49].

None of the pig feeding groups studied displayed an effect from the EMs on intramuscular fat content, which was at a balanced, relatively low level: from 1.66% in the C and E2 groups to 2.45% in the E2 + EM group (Figure 1B). The reason for this may be the selection of pigs towards high meatiness at the expense of lower fat content, and thus, poorer meat quality—including succulence, palatability, and tenderness [8]. Replacing the genetically modified soya bean meal with Albatros peas had a significant impact on reducing the IMF content (from 2.18% and 2.19% to 2.04%) in the *longissimus dorsi* muscle of pigs (*p* < 0.05) [50]. A study by Madera et al. [51] did not demonstrate that diet can have a significant effect on IMF content for either the protein-reduced diet or the control group. Although the authors showed a slight increase in IMF content (from 1.78% to 2.06%) in Alentejana purebred pigs, this is still a small amount. This was clearly confirmed by studies by Lopez et al. [52], who reported a significantly higher intramuscular fat content with a 5% reduction in protein levels in the diet (*p* < 0.003). According to research by Olivares et al. [53], the addition of high levels of vitamin A (dVitA; 100,000 IU) to the diet of Duroc boars resulted in a 20% increase in the IMF of their offspring.

This study did not find any influence of an EM Bokashi preparation on the proportion of properly built muscle cells or the occurrence of giant fibers and necrosis. Normal fibers were found in 95.95% of cases in the E1 group and in 98.27% in the E1 + EM group, which is consistent with the results from other researchers [47]. The most frequently observed pathological changes included giant fibers (Figure 1C) (from 1.10% in the E1 + EM group to 1.99% in the C group), which can be formed from any type of muscle fiber (STO, FTO, or FTG). According to Wojtysiak [54], their occurrence is dependent on nutrition, the handling of animals before slaughter, genetic conditions, and the breed—especially those with a high susceptibility to stress. The presence of giant fibers has a significant impact on the acidity and texture parameters of meat [54]. According to Górska and Wojtysiak [55], PSE turkey meat contributes to a significant increase in the occurrence of giant fibers in muscle tissue.

Degenerative changes causing the breakdown of muscle fibers (necrosis) are indicated by the appearance of histiocytes, lymphocytes, and macrophages (Figure 1D) [56]. In terms of fiber splitting, the results were inconclusive. Fiber splitting is one of the pathological changes caused by excessive cell overload. It is usually related to coarse fibers and results from hypoxia and low metabolite uptake [57]. The adverse effects of using effective microorganisms were observed in the C + EM control and E2 + EM experimental groups, where splitting was significantly higher (0.68% vs. 1.01% and 0.81% vs. 1.15%, respectively; *p* < 0.05) than in the control groups (Figure 1C). Differing, favourable results were reported in the experimental E1 group. Pigs from this group, fed with the addition of effective microorganisms, were characterized by a significantly lower percentage of fiber splitting (1.45% vs. 0.83%; *p* < 0.05). This is confirmed by previous research by Bogucka et al. [47], who also showed a significant beneficial effect adding a synbiotic to chickens’ diet had on the percentage of split fibers. Despite the inconclusive results of Semenova et al. [58], they recommend the use of feed additives (selenium, vitamin E, flavonoids, etc.) in order to reduce the occurrence of pathological changes, which appears to be decisively linked with the quality of meat.

### 3.3. Percentage of Selected Muscle Fiber Proteins in Longissimus Lumborum Muscle

Table 5 shows the percentage of selected proteins in the *LL* muscle of pigs depending on the nutritional variant used.

**Figure 2:**
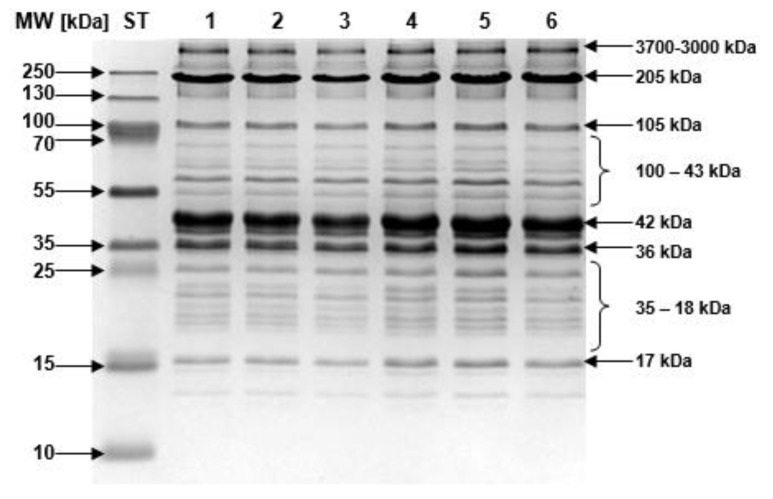
Comparison of distributions of muscle fiber protein types in longissimus lumborum: 1, 3, 5—control groups (C, E1, and E2, respectively); 2, 4, 6 experimental groups (C + EM, E1 + EM, and E2 + EM, respectively).

Additionally, the elecrophoreogram (Figure 2) shows six analyzed protein bands and two ranges in relation to the molecular weight of the standard used. The highest percentage, i.e., from 30% to 33.88%, was characteristic of proteins with a molecular weight of 42 kDa, while the lowest was for proteins with molecular weights of 17 and 105 kDa. Only in the case of proteins in the 3000–3700 kDa range and for the 42 kDa protein, a different feeding system turned out to be a factor determining their quantity. For most proteins, no statistically significant influence of EMs on the percentage of them in the muscle was noted (Table 5). The exceptions were proteins weighing 205 kDa (*p* < 0.05), which showed a significantly higher proportion in the C group compared to C + EM, and proteins with a weight of 36 kDa (*p* < 0.05) between the E1 control group and the E1 + EM experimental group. Results similar to those presented in the study, indicating an influence of supplementation on *LL* muscle protein changes in a lamb diet, were obtained by Malva et al. [59]: based on the protein profile, a decrease in myosin (MHC) after supplementation with quinoa and linseed was observed in their study, as well as a decrease in Tn-T with linseed supplementation. However, Adeyemi et al. [60] did not find any significant changes in the electrophoretic sections of goat meat using rapeseed and palm oil. The changes in the 205 kDa band suggest that supplementation may affect post-mortem degradation processes and may change the integrity of the actomyosin complex. On the other hand, a decrease in this protein type may indicate oxidation processes that may induce conformational and functional modifications of muscle proteins, including the activity of the calpain system, which are enzymes particularly susceptible to oxidation [61,62].

Due to the nature of the experiment conducted, the description of electrophoretic tests also includes statistically significant correlations between the percentage of proteins and the properties of meat quality and microstructure. The most statistically significant relationships (both positive and negative) were recorded between proteins and percentage fat content and percentage of FTO fibers (IIA), as well as some meat quality properties, such as color, pH_48_, tenderness, and sirloin eye area. Such an interaction between characteristics results from intensive/multiplane chemical processes occurring in muscle tissue, which are often a consequence of these relationships. In relation to the proportion of FTO fibers, positive, statistically significant correlations (*p* < 0.05) were observed for proteins with molecular weights in the range of 100–43 kDa (r = 0.557), 36 kDa (r = 0.803), and 35–17 kDa (r = 0.574), which could be closely related to the aerobic and glycolytic properties of these fibers and their structure [63]. However, for the band with a weight of 17 kDa—indicated as myoglobin—the analysis of linear correlation showed a highly significant negative correlation with the percentage of FTO fibers (r = −0.73; *p* < 0.05). FTO fibers contain less myoglobin than STO fibers, which may explain this correlation. Even less of this protein is contained in FTG fibers, but in this case, despite an inversely proportional relationship between the characteristics under consideration, it was statistically insignificant. Muscles composed mainly of type II fast fibers are more susceptible to early post-mortem proteolytic degradation than muscles composed mainly of type I slow fibers [63]. A higher proportion of muscle fibers with a predominant glycolytic metabolism, as obtained in own research, certainly leads to higher levels of glycolytic enzymes. The increased release of myofibrillar proteins in meat into the soluble fraction may indicate more intense proteolysis [64]. The above information is very useful for explaining the relationship between the various parameters obtained in our own research. It is the tenderness, color, taste, and water absorption of the meat that are determined by the proteolytic processes taking place in the muscle tissue after slaughter (*post mortem*) and during meat storage. The proteolytic degradation of myofibrillar and cytoskeletal proteins plays an important role in determining meat tenderness, indicating structural changes in skeletal muscles [65]. In our own research, it is worth noting a negative, statistically significant correlation (r = −0.634; *p* < 0.05) between meat tenderness expressed in cutting force (N/cm^2^) and proteins with a molecular mass between 35 and 18 kDa—corresponding to troponin-T degradation products, among others. Moreover, it is worth noting obtained in own research the relationship between the number and percentage of FTO fibers and proteins with a molecular mass of 105 kDa, whose weight corresponds to alpha-actinin (r = −0.620 and r = −0.789, respectively; *p* < 0.05). A higher percentage of type II fibers with a simultaneously lower concentration of proteins with a weight of 105 kDa which has been demonstrated in own research and less of the other important degradation products of myofibrillar and cytoskeletal proteins may indicate a less intense meat tenderization process [64,65]. As shown by Grześ et al. [65] the meat tenderization process of the longissimus muscle in pigs involves the degradation or release of muscle tissue proteins, in particular those with masses of 3700, 105, and 38 kDa, and a change in the proportion of centrifugal leakage fraction proteins in the 3700–2400 kDa and 38–36 kDa ranges. Troponin T and its degradation products appearing during storage are an indicator of the meat maturation and tenderization process. As a result of the degradation, the structure of the sarcomere is weakened, which may lead to better tenderness [66]. The relationship observed in our own research confirms the increase in tenderness (decrease in cutting force) of meat as the amount of these products increases.

This study also revealed a positive correlation (r = 0.629; *p* < 0.05) between the 205 kDa band, which contained heavy MHC myosin chains, and the percentage of fat content. It should be emphasised that the composition of lipids is one of the main characteristics related to the quality of meat, and that it is influenced by the type of muscle fibers. However, unlike in our own research, these relationships between IMF intramuscular fat content and fatty acid composition and the presence of myosin isoforms in Cinta Senese pigs [67] were not demonstrated.

In addition, for fast twitch fibers in chickens, the proteins that make up the Z-line are more susceptible to early post-mortem proteolytic degradation than in slow twitch fibers [63,68].

## 4. Conclusions

In conclusion, the quality of meat was satisfactory in all groups of pigs examined. The beneficial effect of adding effective microorganisms (EMs) was found in nutritional variant II, where less thermal leakage from meat was found. Additionally, a beneficial effect of the different diet on less thermal leakage and larger loin eye area in the E2 experimental group was demonstrated. The addition of a probiotic to pigs’ diet resulted in thicker muscle fibers, which was particularly evident in the case of the E1 + EM group, where a significant increase in the diameter of fast twitch fibers was noted. In the same group of pigs, EMs led to less fiber fission, which is a common lesion in fast-growing animals. Additionally, in terms of proteins, the above nutritional variant appears to be the most appropriate because it resulted in the most favorable percentage of the individual proteins after EM supplementation in the LL muscle.

## Figures and Tables

**Table 1:** The composition of feeds.

Dietary Value	Control		E1		E2	
	Phase I (30–70 kg)	Phase II (70–115 kg)	Phase I (30–70 kg)	Phase II (70–115 kg)	Phase I (30–70 kg)	Phase II (70–115 kg)
Dry matter, g	877	875	877	875	877	875
Metabolic energy, MJ	13.39	13.11	13.32	13.09	13.32	13.12
Total protein, g	170	159	171	157	171	160
Fat, g	27	20	29	20	29	20
Lysine, g	10.6	9.7	10.6	9.7	10.6	9.7
Calcium, g	5.9	5.8	5.9	5.8	5.9	5.8
Phosphorus, g	5.3	5.2	5.3	5.2	5.3	5.2
Sodium, g	1.7	1.7	1.7	1.7	1.7	1.7
Vitamin A, IU	10.000	10.000	10.000	10.000	10.000	10.000
Vitamin D, IU	2.200	2.200	2.200	2.200	2.200	2.200
Vitamin E, IU	80	80	80	80	80	80
**Composition of the fodder, %:**						
Soy meal 46% BO	16	12	10	4	10	-
Wheat 12%	20	20	20	20	20	20
Barley 12%	35	45	30	41	30	35
Triticale 10%	25.3	20	26	20	26	20
Soybean oil	1	0.3	1.3	0.3	1.3	0.3
Lupine 37%	-	-	7	9	7	12
Pea 21%	-	-	3	3	3	10
PORKOVITAL T PEA 2.5%	2.5	2.5	2.5	2.5	2.5	2.5
SELACID GG DRY 25 BR	0.2	0.2	0.2	0.2	0.2	0.2
Total	100	100	100	100	100	100

**Table 2:** Experiment arrangements.

Control Group (C)				Experimental Group (E1)				Experimental Group (E2)			
C		C + EM		E1		E1 + EM		E2		E2 + EM	
n = 25		n = 25		n = 25		n = 25		n = 25		n = 25	
♂ 13♀ 12		♂ 12 ♀ 13		♂ 12 ♀ 13		♂ 13 ♀ 12		♂ 13 ♀ 12		♂ 12 ♀ 13	
**Body Weight at Slaughter, kg**											
♂	♀	♂	♀	♂	♀	♂	♀	♂	♀	♂	♀
111.27 ± 5.70	107.70 ± 8.45	113.42 ± 4.94	112.04 ± 3.07	115.63 ± 4.67	109.00 ± 7.29	115.86 ± 4.43	112.50 ± 4.21	113.82 ± 4.88	111.55 ± 4.57	115.86 ± 4.64	115.56 ± 4.30

Group C—standard diet based on post-extraction soya bean meal (100%); groups E1 and E2—in phase I of fattening, a feed mix with 50% soya bean and 50% pea and lupine was administered; group E1—in phase II of fattening a feed mix with 25% soya bean and 75% pea and lupine was administered; group E2—100% of soya bean protein was replaced with pea and lupine; experimental groups, with the addition of a probiotic to the diets (C + EM, E1 + EM, E2 + EM) [14].

**Table 3:** Effects of effective microorganisms (EM) on meat quality in pigs fed on diets with proteins of different origin.

Traits	C		E1		E2	
	C	C + EM	E1	E1 + EM	E2	E2 + EM
pH_48_	5.14 ± 0.12	5.09 ± 0.09	5.13 ± 0.06	5.19 ± 0.10	5.11 ± 0.05	5.13 ± 0.07
WHC, loose water %	15.57 ^a^ ± 2.49 *p* = 0.016	18.16 ^b^ ± 2.50 *p* = 0.016	17.77 ± 2.13	17.01 ± 1.70	18.89 ± 3.72	16.28 ± 1.88
Thermal leakage, %	24.39 ± 1.50	24.25 ± 2.54	24.44 ± 0.63	24.80 * ± 1.96	24.80 ^a^ ± 2.50 *p* = 0.024	22.38 ^b^* ± 1.25 *p* = 0.024
L*—Lightness	56.30 ± 1.92	57.59 ± 3.48	56.49 ± 2.36	55.65 ± 2.55	56.13 ± 1.15	56.39 ± 2.52
a*—Redness	13.91 ± 0.66	14.13 ± 0.86	13.99 ± 1.04	14.41 ± 0.56	14.21 ± 0.78	14.00 ± 0.98
b *—Yelowness	4.50 ± 0.64	4.25 ± 0.77	4.27 ± 0.96	4.51 ± 0.80	4.89 ± 0.55	4.56 ± 0.91
Marbling, score	1.87 ± 0.79	1.47 ± 0.50	1.59 ± 0.49	1.70 ± 0.60	1.39 ± 0.48	1.55 ± 0.51
Tenderness, N	47.94 ± 2.80	46.83 * ± 5.74	47.27 ^a^ ± 7.62 *p* = 0.030	56.27 ^b^* ± 7.28 *p* = 0.030	53.57 ± 5.78	51.34 * ± 5.03
Loin eye area, cm^2^	53.10 ± 5.67	55.76 ± 6.15	53.41 ± 4.45	52.62 * ± 2.21	57.59 ± 4.96	57.93 * ± 3.31

Group C—standard diet based on post-extraction soya bean meal (100%); groups E1 and E2—in phase I of fattening, a feed mix with 50% soya bean and 50% pea and lupine was administered; group E1—in phase II of fattening a feed mix with 25% soya bean and 75% pea and lupine was administered; group E2—100% of soya bean protein was replaced with pea and lupine; experimental groups with the addition of a probiotic to diets (C + EM, E1 + EM, and E2 + EM); ^a, b^ statistically significant differences between two experimental groups within specific nutritional systems at *p* ≤ 0.05; * statistically significant differences between the feeding systems (C vs. E1vs. E2 and C + EM vs. E1 + EM vs. E2 + EM) at *p* ≤ 0.05; *p*-value for groups that differ statistically significantly within one feeding system.

**Table 4:** Effects of effective microorganisms (EM) on the microstructure of *longissimus lumborum* muscle in pigs fed on diets with proteins of different origin.

Traits		C		E1		E2	
		C	C + EM	E1	E1 + EM	E2	E2 + EM
Percentage of muscle fibers, %	STO	13.60 ± 2.50	16.33 ± 6.60	14.36 ± 4.68	14.51 ± 3.60	16.10 ± 4.41	15.41 ± 4.77
	FTO	13.69 ± 2.82	13.52 ± 3.57	13.80 ± 2.40	14.05 ± 3.27	14.70 ± 3.76	15.13 ± 2.20
	FTG	72.71 ± 3.21	70.15 ± 9.25	71.84 ± 6.35	71.44 ± 3.49	69.20 ± 16.14	69.46 ± 20.62
Fiber diameter, µm	STO	35.03 ± 4.34	35.86 ± 4.69	38.86 ± 5.61	40.82 ± 5.21	37.11 ± 3.38	39.53 ± 2.65
	FTO	35.44 ± 6.52	40.66 ± 6.63	35.78 ^a^ ± 3.88 *p* = 0.003	43.17 ^b^ ± 3.51 *p* = 0.003	37.24 ^a^ ± 5.46 *p* = 0.028	39.42 ^b^ ± 2.26 *p* = 0.028
	FTG	47.14 ± 4.50	52.30 ± 8.71	44.68 ^a^* ± 5.16 *p* = 0.012	50.57 ^b^ ± 3.46 *p* = 0.012	51.26 * ± 2.63	52.27 ± 3.26
Muscle fiber density (fiber number/1,5 mm^2^)	Sum	244 ± 48	228 ± 67	243 ± 60	205 ± 22	222 ± 20	232 ± 23
	STO	35 ± 10	34 ± 9	33 ± 7	30 ± 8	34 ± 10	28 ± 10
	FTO	34 ± 10	31 ± 11	33 ± 7	29 ± 7	27 ± 8	31 ± 7
	FTG	175 ± 34	163 ± 65	177 ± 52	146 ± 16	161 ± 13	173 ± 21
Total number of muscle fibers (×1000) (TNF)		744 ^a^ ± 368 *p* = 0.044	694 ^b^ ± 488 *p* = 0.044	743 ± 371	617 ± 254	660 ± 406	652 ± 439
Intramuscular fat content, (histochemically) %		1.66 ± 1.55	2.27 ± 1.13	1.84 ± 1.21	1.67 ± 0.96	1.66 ± 2.05	2.45 ± 1.79
Normal fibers, %		97.22 ± 2.02	97.36 ± 1.97	95.95 ± 2.72	98.27 ± 1.02	96.72 ± 1.71	96.60 ± 1.86
Giant fibers, %		1.99 ± 1.84	1.29 ± 1.29	1.76 ± 1.80	1.10 ± 1.17	1.87 ± 1.16	1.95 ± 1.50
Fiber necrosis, %		0.11 ± 0.18	0.34 ± 0.53	0.84 ± 3.03	0.80 ± 0.77	0.60 ± 0.82	0.30 ± 0.46
Fiber splitting, %		0.68 ^a^* ± 0.25 *p* = 0.041	1.01 ^b^ ± 0.92 *p* = 0.041	1.45 ^a^* ± 1.1 *p* = 0.024	0.83 ^b^ ± 0.81 *p* = 0.024	0.81 ^a^* ± 0.77 *p* = 0.040	1.15 ^b^ ± 0.82 *p* = 0.040

Group C—standard diet based on post-extraction soya bean meal (100%); groups E1 and E2—in phase I of fattening, a feed mix with 50% soya bean and 50% pea and lupine was administered; group E1—in phase II of fattening a feed mix with 25% soya bean and 75% pea and lupine was administered; group E2—100% of soy bean protein was replaced with pea and lupine; experimental groups with the addition of a probiotic to diets (C + EM, E1 + EM, E2 + EM); STO—slow twitch oxidative fibers; FTO—fast twitch oxidative fibers, FTG—fast twitch glycolytic fibers; TNF—total number fibers; ^a, b^ statistically significant differences between two experimental groups within specific nutritional systems at *p* ≤ 0.05; * statistically significant differences between the feeding systems (C vs. E1 vs. E2 and C + EM vs. E1 + EM vs. E2 + EM) at *p* ≤ 0.05; p-value for groups that differ statistically significantly within one feeding system

**Table 5:** Percentage of muscle proteins in longissimus lumborum obtained through electrophoretic separation on polyacrylamide gels with SDS (SDS-PAGE).

Proteins Molecular Weight (kDa)	C		E1		E2	
	C	C + EM	E1	E1 + EM	E2	E2 + EM
3000–3700 titin	7.407 *± 0.428	7.385 * ± 0.431	6.152 * ± 0.836	5.785 * ± 1.051	6.708 ± 1.173	6.832 ± 0.760
205 myosin and titin degradation products	18.118 ^a^ ± 0.385 *p* = 0.027	17.102 ^b^ ± 0.883 *p* = 0.027	17.118 ± 1.171	15.807 ± 1.789	18.060 ± 0.892	17.912 ± 2.195
105 alpha actinin	5.402 ± 1.75	6.022 ± 0.406	5.732 ± 1.654	5.365 ± 1.496	6.627 ± 0.624	6.382 ± 1.017
100–43(kDa)	6.070 ± 0.492	6.305 ± 0.896	6.112 ± 0.552	7.047 ± 0.905	6.588 ± 0.796	6.920 ± 0.578
42 actin	32.207 ± 3.048	31.270 ± 1.596	33.010 ± 2.380	33.885 * ± 3.045	30.063 ± 2.294	30.002 * ± 2.476
36 troponin-T	10.057 ± 0.790	10.783 ± 0.646	10.568 ^b^ ± 0.389 *p* = 0.015	11.172 ^a^ ± 1.296 *p* = 0.015	9.700 ± 1.196	9.918 ± 1.142
35- 18 including troponin-T degradation products	9.092 ± 0.877	9.287 ± 0.776	9.563 ± 0.727	9.525 ± 1.599	9.492 ± 2.204	9.392 ± 1.215
17 myoglobin	5.478 ± 2.378	5.282 ± 0.823	5.365 ± 0.936	5.4383 ± 1.189	6.110 ± 0.834	6.347 ± 1.513

Group C—standard diet based on post-extraction soya bean meal (100%); groups E1 and E2—in phase I of fattening, a feed mix with 50% soya bean and 50% pea and lupine was administered; group E1—in phase II of fattening a feed mix with 25% soya bean and 75% pea and lupine was administered; group E2—100% of soya bean protein was replaced with pea and lupine; experimental groups fed an additional probiotic in the diets (C + EM, E1 + EM, E2 + EM; ^a, b^ statistically significant differences between two experimental groups within specific nutritional systems at *p* ≤ 0.05; * statistically significant differences between the feeding systems (C vs. E1vs. E2 and C + EM vs. E1 + EM vs. E2 + EM) at *p* ≤ 0.05; *p*-value for groups that differ statistically significantly within one feeding system.
